# Efficacy and safety of combined endoscopic rubber band ligation in the treatment of grade II-III prolapsed hemorrhoids: a retrospective study (with video)

**DOI:** 10.3389/fmed.2026.1755971

**Published:** 2026-03-25

**Authors:** Taiyu Chen, Guang Cao, Min Zhong, Foqiang Liao, Jitao Ao, Feng Gao, Huanqi Song, Yingying Zhong, Xu Shu, Hu Chen

**Affiliations:** 1 Department of Gastroenterology, Jiangxi Provincial Key Laboratory of Digestive Diseases, Jiangxi Clinical Research Center for Gastroenterology, Digestive Disease Hospital, The First Affiliated Hospital, Jiangxi Medical College, Nanchang University, Nanchang, Jiangxi, China; 2 Department of Gastroenterology, The First Affiliated Hospital of Yichun University, People's Hospital of Wanzai County, Wanzai, Jiangxi, China; 3 Yichun Clinical Medical Research Center for Gastrointestinal Diseases, Wanzai, Jiangxi, China; 4 Department of gynecology and obstetrics, Wanzai County Hospital of Traditional Chinese Medicine, Wanzai, Jiangxi, China

**Keywords:** efficacy, endoscopic rubber band ligation, Endoscopy, Hemorrhoids, Internal hemorrhoids

## Abstract

**Background:**

Hemorrhoids is one of the most common anorectal diseases. In recent years, the incidence rate of hemorrhoids has been rising.

**Aims:**

To investigate the efficacy and safety of combined endoscopic rubber band ligation (ERBL) in the treatment of grade II-III prolapsed internal hemorrhoids.

**Methods:**

Patients with grade II-III prolapsed internal hemorrhoids treated from January 2019 to June 2023 were reviewed.

**Results:**

Complete resolution was achieved in 124 cases (91.85%), partial resolution in 7 cases (5.19%), and recurrence in 4 cases (2.96%). 34 (25.19%) cases experienced anal pain after procedure, with a median visual analog scale (VAS) score of 0 (0–3). Postoperative bleeding occurred in 18 (13.33%) cases. Anal swelling was observed in 19 (14.07%) cases, and urinary retention occurred in 4 cases (2.96%). The incidence of postoperative pain (30.43% vs. 11.6%, *P* = 0.018) and VAS scores (0 vs. 0, *P* = 0.003) in the ligate three hemorrhoids group were significantly higher compared to the ligate two hemorrhoids group. There was no significant difference in long-term efficacy between the two groups. Male [odds ratio (OR) 5.654, 95% confidence interval (CI) 1.222–26.159; *P* = 0.027] and postoperative complications (OR 4.914, 95% CI 1.080–22.345; *P* = 0.039) were independent risk factors that were incomplete resolution.

**Conclusions:**

Combined ERBL is an efficient and safe method for grade II-III prolapsed internal hemorrhoids. The number of hemorrhoids ligated had no significant effect on the long-term outcome, but the ligation of three hemorrhoids was associated with an increased incidence of postoperative pain. Male and postoperative complications are risk factors for incomplete resolution.

## Introduction

Hemorrhoids, as one of the most common anorectal diseases, account for approximately 98% of all anorectal diseases in China, with a prevalence rate of about 50% (1). Current interventions mainly include non–procedural (dietary and behavioral modifications and medical treatment), instrumental [rubber band ligation (RBL), sclerotherapy and infrared coagulation] and surgical treatments (2, 3). With the continuous development of endoscopic technology, endoscopic treatment of internal hemorrhoids has gradually become popular. Compared with traditional instrumental therapy under rigid proctoscopy, endoscopic therapy is more flexible and has a clearer field of view (4). In China, the endoscopic treatment for internal hemorrhoids is mainly endoscopic rubber band ligation (ERBL) and endoscopic sclerotherapy, of which ERBL is better than endoscopic sclerotherapy for patients with severe prolapse symptoms (5). ERBL has three location options of ligation: hemorrhoid ligation, hemorrhoid proximal mucosa ligation and hemorrhoid and proximal mucosa combined ligation (5). However, few studies have investigated the difference in efficacy between different ligation location, and the effect of the number of hemorrhoids to ligate and the number of bands used on the efficacy (6). Combined ligation is an effective method, but may increase the incidence of postoperative pain (7–9). It has been suggested to avoid ligating 3 hemorrhoids once to reduce postoperative discomfort, but one study suggested that increasing the number of ligation bands could increase the efficacy of grade III hemorrhoids (6, 10). In addition, identifying risk factors for incomplete resolution is critical to improving patient treatment strategies. This study aimed to evaluate the safety and efficacy of combined endoscopic rubber band ligation in the treatment of grade II–III prolapsed internal hemorrhoids, investigate the impact of the number of ligated hemorrhoids on therapeutic outcomes and complications, and identify risk factors for incomplete resolution.

## Methods

### Patients and data collection

Patients receiving endoscopic minimally invasive treatment for stage II and III hemorrhoids in the department of gastroenterology, the First Affiliated Hospital of Yichun University (People's Hospital of Wanzai County) from January 1, 2019 to June 30, 2023 were selected. According to the number of hemorrhoids were ligated, the patients were divided into ligate two hemorrhoids group and ligate three hemorrhoids group. The ligate two hemorrhoids group mainly consisted of patients from 2019 to 2022, and the patients after 2022 were basically ligated three hemorrhoids. Patients with complications (incarcerated hemorrhoids, thrombotic hemorrhoids, etc.), continuous use of anticoagulants, severe organ failure, anal fistula, coagulation dysfunction, mental illness, pregnancy, and a history of colorectal malignancy or anorectal surgery were also excluded. This study was approved by the Ethics Committee of the First Affiliated Hospital of Yichun University (People's Hospital of Wanzai County) (No. 2025001) and was performed in accordance with the Declaration of Helsinki. Written informed consent form was waived.

### Procedures

The entire procedure process employed gastroscopes (EG27-i10, EG2990i; HOYA Corporation, PENTAX Lifecare Division, Tokyo, Japan). Combined ERBL procedure: A 7 Shooter Saeed Multi-band Ligator (M00542251; Boston Scientific Corporation, Natick, MA, USA) was installed at the tip of the gastroscope. In the first step, the normal mucosa above the hemorrhoidal nucleus was ligated. The ligation point was approximately 3–4 cm above the dentate line. Under the inverted view, negative pressure aspiration was performed on the mucosa until the transparent cap was filled and the entire field of vision turned red, followed by the release of the ligation ring by rotating the handle of the ligation device. In the second step, the hemorrhoidal nucleus was ligated under the direct view, and the ligation point was approximately 1 cm above the dentate line. Postoperatively, patients were prescribed a liquid diet based on the physician's experience. In the event that patients experienced discomfort in the anal area, they were advised to take warm sitz baths. If the bleeding did not stop spontaneously, anti-inflammatory and hemostatic ointments were applied topically for cases with a small amount of bleeding, and secondary endoscopic treatment or surgical intervention was contemplated for cases with a large amount of bleeding. If they had intolerable pain, analgesics (aubucaine gel) were prescribed for external use. If patients developed urinary retention, local hot compresses were provided for mild cases, and catheterization was carried out for severe cases. If patients developed thrombotic external hemorrhoids, anti-inflammatory and analgesic ointments were applied topically for mild pain cases, and surgical intervention was considered as appropriate for severe cases.

### Evaluations before and after the procedure

The bleeding score, hemorrhoid disease symptom score (HDSS), and quality of life score were used to evaluate the severity of preoperative symptoms (11). During the postoperative hospital stay, complications within 1 week after the operation were observed to evaluate the safety of the procedure. Postoperative pain was assessed using the visual analog scale (VAS).

### Long-term follow-up and outcome assessment

The long-term effect, recurrence of hemorrhoids and improvement of postoperative complications were observed through outpatient or telephone follow-up. There are three levels of efficacy: (1) Complete resolution: the patient's symptoms disappear completely; (2) Partial resolution: symptoms improve from before treatment; and (3) Ineffective: symptoms do not improve or worsen. Partial resolution and inefficiencies are considered incomplete resolution. HDSS and quality of life score were used to evaluate the severity of symptoms.

### Statistical analysis

Data are expressed by number (*n*) or rate (%). Data with normal distribution were expressed as mean ± standard deviation (x ± s), and those with abnormal distribution were expressed as median and interquartile range. Quantitative variables were compared using the independent *t* test or Mann–Whitney U test between two groups. Chi-square test or Fisher exact test were used for the comparisons of qualitative variables. Univariate and multivariate logistic regression analysis were used to investigate the risk factors of incomplete resolution.

SPSS Statistics (version 23.0; IBM, Armonk, New York, USA) was utilized for data analysis. A value of *P* < 0.05 was considered statistically significant.

## Results

### Characteristics and outcomes of the total patients

**Figure 1:**
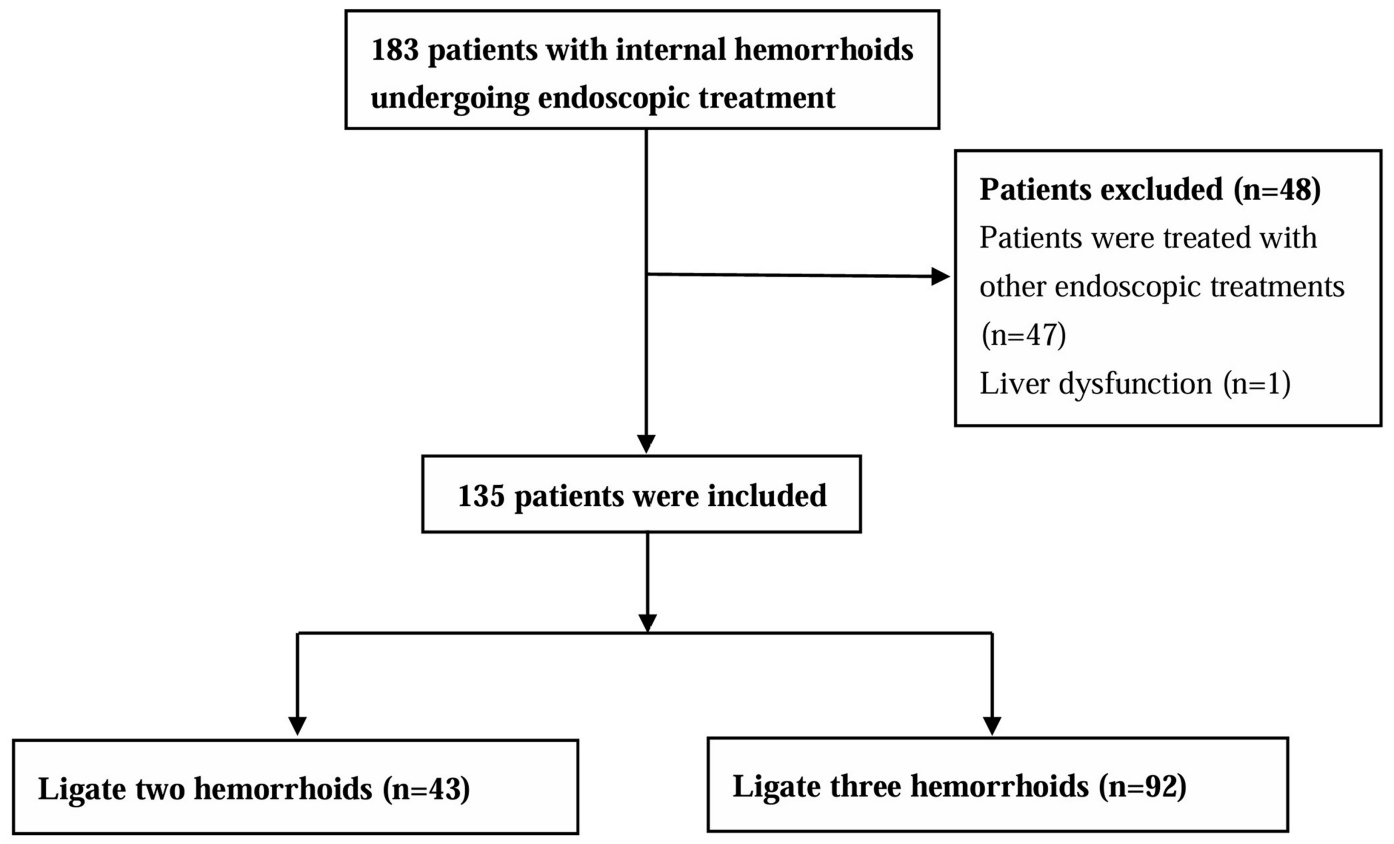
The flowchart of patients included in the study.

**Figure 2:**
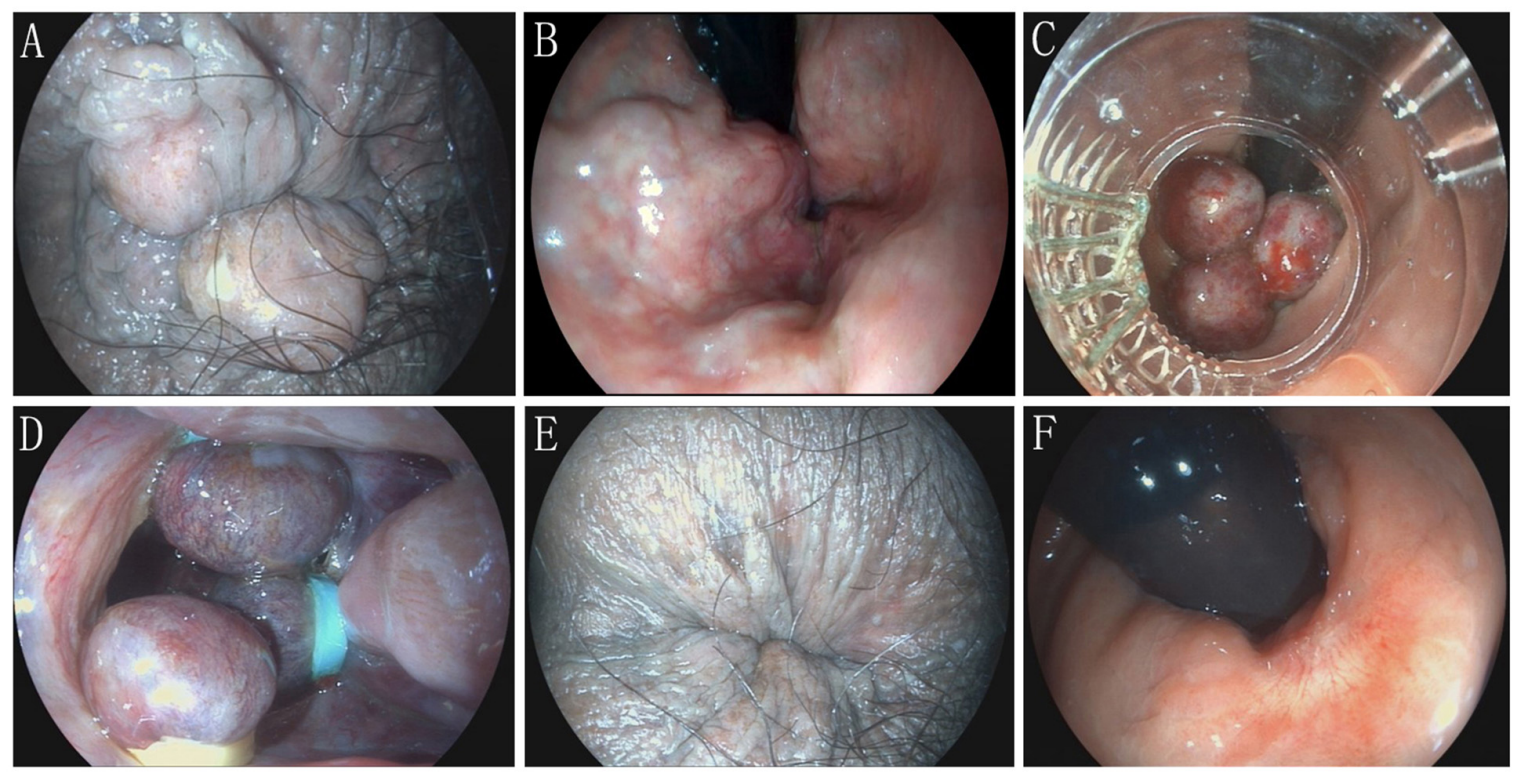
Combined endoscopic rubber band ligation in the treatment of a patient with grade III internal hemorrhoids. **A**. Preoperative external anal manifestations; **B**. Preoperative anal canal manifestations; **C**. Ligation of normal mucosa at the proximal end of hemorrhoids; **D**. Ligation of three hemorrhoids; **E**. Postoperative external anal manifestations; **F**. Anal canal manifestations 6 months after the procedure.

A total of 135 patients were enrolled after screening according to inclusion and exclusion criteria, and all patients completed follow-up (Figure 1). The mean age of all patients at baseline was 53.12 ± 10.71 years, including 46 males (34.1%) and 89 females (65.9%). The mean BMI was 23.45 ± 3.39 kg/m^2^. All patients had prolapsed symptoms, 34 (25.2%) with bleeding, 8 (5.9%) with pain, and 66 (48.89%) with pruritus. 69 (51.11%) were diagnosed with Goligher grade II and 66 (48.89%) with Goligher grade III. None of the patients had undergone colorectal surgery before. The median bleeding score was 0 (0–2), the median hemorrhoid symptom score was 3 (3–5), and the median quality of life score was 2 (1–3) (Table 1). Figure 2 shows an example of endoscopic images before and after combined ERBL treatment. Within 7 days after ERBL procedure, 34 (25.19%) cases experienced anal pain, with a median VAS score of 0 (0–3). Analgesics were required in 25 (18.52%) cases. Postoperative bleeding occurred in 18 (13.33%) cases, none of which necessitated endoscopic therapy. Anal swelling was observed in 19 (14.07%) cases, and urinary retention occurred in 4 cases (2.96%). During long-term follow-up spanning a median of 29 (12–34) months, complete resolution was achieved in 124 cases (91.85%), partial resolution in 7 cases (5.19%), and recurrence in 4 cases (2.96%) (Table 2).

**Table 1:** Clinical characteristics of the total cohort.

**Characteristic**	**Combined ERBL group (N = 135)**
**Gender (** ***n*** **) (%)**	
Male	46 (34.07)
Female	89 (65.93)
Age (years) (mean ± SD)	53.12 ± 10.71
BMI (kg/m^2^) (mean ± SD)	23.45 ± 3.39
**Goligher grade (** ***n*** **) (%)**	
II	69 (51.11)
III	66 (48.89)
**Symptom (** ***n*** **) (%)**	
Prolapse	135 (100)
Bleeding	34 (25.19)
Pain	8 (5.93)
Anal pruritus	9 (6.67)
Bleeding score, median (IQR)	0 (0–2)
Hemorrhoidal disease symptom score, median (IQR)	3 (3–5)
Quality of life score, median (IQR)	2 (1–3)

**Table 2:** Postoperative complications and long-term outcomes of the total cohort.

**Characteristic**	**Combined ERBL group (N = 135)**
**Postoperative complications (**<**7 days)**	
**Postoperative pain (** ***n*** **) (%)**	
Yes	34 (25.19)
No	101 (74.81)
Pain VAS, median (IQR)	0 (0–3)
**Analgesic requirement (** ***n*** **) (%)**	
Yes	25 (18.52)
No	110 (81.48)
**Postoperative bleeding (** ***n*** **) (%)**	
Yes	18 (13.33)
No	117 (86.67)
**Urinary retention (** ***n*** **) (%)**	
Yes	4 (2.96)
No	131 (97.04)
**Anal swelling (** ***n*** **) (%)**	
Yes	19 (14.07)
No	116 (85.93)
**Long-term outcomes**	
Hemorrhoidal disease symptom score, median (IQR)	0 (0–0)
Quality of life score, median (IQR)	0 (0–0)
**Procedure outcomes (** ***n*** **) (%)**	
Complete resolution	124 (91.85)
Partial resolution	7 (5.19)
Ineffective	4 (2.96)
Retreatment	5 (3.70)

### Comparison between the two groups

The patients were divided into ligate two hemorrhoids group (N = 43) and ligate three hemorrhoids group (N = 92). There were no significant differences in baseline information between the two groups (Table 3). The incidence of postoperative pain (11.6% vs. 30.43%, respectively; *P* = 0.018) and VAS scores [0 (0–1) vs. 0 (0–3.75), respectively; *P* = 0.003] in the ligate three hemorrhoids group were significantly higher compared to the ligate two hemorrhoids group. The analgesic usage rate in the ligate three hemorrhoids group was higher than that in the ligate two hemorrhoids group, but the difference was not statistically significant (9.30% vs. 22.83%, respectively; *P* = 0.059). There were no significant differences in the incidence of postoperative bleeding (13.95% vs. 13.04%, respectively; *P* = 0.885), urinary retention (2.32% vs. 3.26%, respectively; *P* = 1.000) and anal swelling (13.95% vs. 14.13%, respectively; *P* = 0.978) between the two groups. Hemorrhoidal disease symptom score [0 (0–0) vs. 0 (0–0), *P* = 0.334] and quality of life score [0 (0–0) vs. 0 (0–0), *P* = 0.355] had no significant difference between the two groups There was no significant difference in the long-term outcomes between the two groups (*P* = 0.427). In the 2 hemorrhoids ligated group, 1 patient received re-treatment, and in the 3 hemorrhoids ligated group, 4 patients received re-treatment. There was no significant difference in the re-treatment rate (2.33% vs. 4.35%, *P* = 0.566) (Table 4).

**Table 3:** Clinical characteristics of the two groups.

**Characteristic**	**Ligate 2 hemorrhoids (N = 43)**	**Ligate 3 hemorrhoids (N = 92)**	***P* value**
**Gender (** ***n*** **) (%)**			
Male	13 (30.23)	33 (35.87)	0.520
Female	30 (69.77)	59 (64.13)	
Age (years) (mean ± SD)	53.65 ± 10.75	52.88 ± 10.74	0.698
BMI	22.98 ± 3.60	23.67 ± 3.28	0.274
**Goligher grade (** ***n*** **) (%)**			
II	25 (58.14)	44 (47.83)	0.264
III	18 (41.86)	48 (52.17)	
Hemorrhoidal disease symptom score, median (IQR)	3 (3–5)	3 (3–5)	0.574
Bleeding score, median (IQR)	0 (0–2)	0 (0–0)	0.930
Quality of life score, median (IQR)	1 (1, 2)	2 (1–3)	0.175

**Table 4:** Postoperative complications and long-term outcomes of the two groups.

**Characteristic**	**Ligate 2 hemorrhoids (N = 43)**	**Ligate 3 hemorrhoids (N = 92)**	***P* value**
**Postoperative complications (**<**7 days)**			
**Postoperative pain (** ***n*** **) (%)**			
Yes	5 (11.63)	28 (30.43)	**0.018**
No	38 (88.37)	64 (69.57)	
Pain VAS, median (IQR)	0 (0–1)	0 (0–3.75)	**0.003**
**Analgesic requirement (** ***n*** **) (%)**			
Yes	4 (9.30)	21 (22.83)	0.059
No	39 (90.70)	71 (77.17)	
**Postoperative bleeding (** ***n*** **) (%)**			
Yes	6 (13.95)	12 (13.04)	0.885
No	37 (86.05)	80 (86.96)	
**Urinary retention (** ***n*** **) (%)**			
Yes	1 (2.32)	3 (3.26)	1.000
No	42 (97.67)	89 (96.74)	
**Anal swelling (** ***n*** **) (%)**			
Yes	6 (13.95)	13 (14.13)	0.978
No	37 (86.05)	79 (85.87)	
**Long-term outcomes (**>**12 months)**			
Hemorrhoidal disease symptom score, median (IQR)	0 (0–0)	0 (0–0)	0.334
Quality of life score, median (IQR)	0 (0–0)	0 (0–0)	0.355
**Procedure outcomes (** ***n*** **) (%)**			
Complete resolution	40 (93.02)	84 (91.30)	0.427
Partial resolution	1 (2.33)	6 (6.52)	
Recurrence	2 (4.65)	2 (2.17)	
Retreatment	1 (2.33)	4 (4.35)	0.566

### Risk factors for predict the incomplete resolution

Univariate and multivariate regression analyses found that male [odds ratio (OR) 5.654, 95% confidence interval (CI) 1.222–26.159; *P* = 0.027] and short-term postoperative complications [OR 4.914, 95% (CI) 1.080–22.345; *P* = 0.039] were independent risk factors that were incomplete resolution (Table 5).

**Table 5:** Risk factors for predict the incomplete resolution.

**Factors (*n*)**	**Univariable analysis**	**Multivariable analysis**
	**OR (95%CI)**, ***P*** **value**	**aOR (95%CI)**, ***P*** **value**
**Gender (** ***n*** **)**		
Female (7)	1 (Reference)	1 (Reference)
Male (4)	3.675 (1.017–13.282), **0.047**	5.654 (1.222–26.159), **0.027**
Age (years)	0.969 (0.914–1.028), 0.299	0.962 (0.900–1.027), 0.243
BMI	0.939 (0.776–1.138), 0.522	0.905 (0.744–1.102), 0.321
**Goligher grade**		
II (7)	1 (Reference)	1 (Reference)
III (6)	0.750 (0.293–1.918), 0.548	1.453 (0.139–15.232), 0.755
**Ligate hemorrhoids**		
2 hemorrhoids (3)	1 (Reference)	1 (Reference)
3 hemorrhoids (8)	1.280 (0.371–4.414), 0.696	1.494 (0.283-7.889), 0.636
Hemorrhoidal disease symptom score	0.933 (0.596-1.459), 0.760	0.252 (0.014–3.071), 0.252
Bleeding score	1.184 (0.871–1.609), 0.280	2.781 (0.570–13.562), 0.206
Quality of life score	1.187 (0.652–2.163), 0.575	1.049 (0.103–10.653), 0.968
**Postoperative complications**		
No (3)	1 (Reference)	1 (Reference)
Yes (8)	3.692 (0.934–14.589), 0.062	4.914 (1.080–22.345) **0.039**

## Discussion

The pathogenesis of hemorrhoids is not clear at present, the mainstream view is the abnormal sliding of the anal pad in the anal canal (35, 36). Different ideas about the pathogenesis of hemorrhoids have led to different surgical methods (36). In order to reduce the burden on patients, non-surgical treatment is generally used for low-grade hemorrhoids, and surgical treatment is chosen when high-grade hemorrhoids or non-surgical treatment fails (37). In recent years, the purpose of treating hemorrhoids has changed from completely eliminating hemorrhoids to eliminating symptoms (5, 38). Although surgical treatment is more effective than non-surgical treatment, it comes at the cost of more pain and complications (12–14, 39). For grade I-III internal hemorrhoids, office management may be more cost-effective. Sclerotherapy and RBL are the most common office therapies in China. Sclerotherapy is more convenient to operate, and the incidence of pain is lower than RBL, but RBL is more effective than sclerotherapy (9, 15–17). For patients with hemorrhoids whose main symptom is prolapse, RBL may be a better option (18).

The main principle of RBL is to promote rectal mucosal fibrosis and reduce blood flow to hemorrhoids to prevent prolapse and bleeding (19). RBL performed under proctoscopy can only provide a limited field of view and it is difficult to perform multiple ligations at once. ERBL is flexible and visualized in operation, avoiding blind ligation. Moreover, it is convenient to perform multiple ligations with the ligation device used for treating gastroesophageal varices. A randomized controlled trial found that RBL and ERBL had similar efficacy and safety, but ERBL required fewer treatment sessions (20). The ligation site in ERBL is typically selected at either the hemorrhoids or the normal rectal mucosa proximal to the hemorrhoids. The combined ERBL technique simultaneously ligates both the normal rectal mucosa proximal to hemorrhoids and the hemorrhoids themselves, aiming to enhance therapeutic efficacy for prolapse.

In this study, the complete resolution rate reached 91.85%, which was close to the previous studies (7, 8). During the long-term follow-up, the symptoms of 124 patients were completely relieved, the symptoms of 7 patients partially improved, and the symptoms of 4 patients did not improve or recurred. Five patients underwent retreatment within 1 to 6 months after the procedure. Among them, 4 patients underwent endoscopic treatment again and 1 patient underwent operative treatment.

In terms of safety, common complications of ERBL include bleeding, thrombosis of external hemorrhoids, anal discomfort and urinary retention. Severe complications include massive hemorrhage, pelvic sepsis, liver abscess, etc. Massive bleeding is generally considered to be caused by premature detachment of the ligation band or the patient's recent use of anticoagulants (21–23). Pelvic sepsis and liver abscess have been reported in cases treated with RBL, but not in cases with ERBL (24–27). In this study, no patients had severe postoperative complications, and all postoperative complications were relieved during the hospital observation period. The total incidence of postoperative pain was 25.19%. Two previous randomized controlled studies respectively found that 74.2% and 27% of patients with combined ERBL experienced postoperative pain. The difference in the data might be due to the different criteria for judging postoperative pain in different studies (7, 8). Twenty-five patients (18.52%) who could not tolerate anal discomfort all had their pain relieved after being given analgesic. Eighteen patients (13.33%) had mild bleeding within 7 days after the procedure, and no endoscopic hemostasis was required. Urinary retention occurred in 4 patients (8.1%), and their urinary function recovered within 3 days after hot compress.

There is no unified standard or consensus for the number of bands used and the number of hemorrhoids to ligate in a single session of ERBL. The guidelines in China only recommend that no more than 7 ligation bands be used in a single session, for grade I-II internal hemorrhoids, it is advisable to avoid banding all hemorrhoids simultaneously (5). Some suggest that a single treatment should avoid banding three hemorrhoids simultaneously, However, other studies have found that ligating three hemorrhoids at once can shorten the treatment process and the complications are acceptable (10, 28–30). This study found that the incidence of pain and VAS score in the ligate three hemorrhoids group were significantly higher than those in the ligate two hemorrhoids group. The analgesic requirement rate in the ligate three hemorrhoids group (22.83%) was higher than that in the ligate two hemorrhoids group (9.30%), but the difference was not significant. This might be because ligating three hemorrhoids requires more ligation bands, increasing the discomfort of patients (31). Long-term follow-up found no significant difference in efficacy between the two groups, suggesting that combined ERBL may not require to ligate three hemorrhoids once.

Multivariate regression analysis revealed that male gender and postoperative complications were independent risk factors leading to incomplete resolution. One study has found that male gender is significantly associated with the severity and recurrence of hemorrhoids, other studies have found that male gender is a risk factor for delayed bleeding and readmissions after hemorrhoidectomy, which may be attributed to their higher levels of physical activity (32–34, 40). We found that short-term complications after procedure are also independent risk factors for incomplete resolution. Some of the bleeding may be due to incomplete ligation, and bleeding and anal discomfort may cause patients to be reluctant to defecate, leading to constipation and thereby increasing the risk of recurrence of hemorrhoids (32).

The study still has several limitations. Firstly, this is a single-center retrospective study, information bias is unavoidable. In particular, treatment assignments were nonrandomized and subject to potential historical bias. However, in this study, the group expected to have more mature techniques (the group with three hemorrhoids ligation) had a significantly higher incidence of pain and VAS score, and there was no significant difference in long-term efficacy. Therefore, the increase in ligation bands may be a more direct cause of postoperative complications rather than the learning curve of endoscopists. This bias has a limited impact on the results. However, prospective randomized studies are needed to overcome this limitation in the future. Secondly, as a retrospective study, a formal sample size calculation was not performed prior to data collection. However, the sample size of 135 patients is comparable to or larger than that of several previously published studies on combined ERBL (7, 8), suggesting adequate power for the primary analyses of efficacy and safety. In addition, it should be noted that the sample size for incomplete resolution was small and the statistical power of the regression analysis was limited. Therefore, the results for risk factors should be interpreted with caution, mainly to provide hypotheses for future prospective studies with larger samples.

## Conclusion

In conclusion, combined endoscopic rubber band ligation is an efficient and safe method for grade II-III internal hemorrhoids with prolapse as the main symptom. The number of hemorrhoids ligation had no significant effect on the outcome, but ligation of only two hemorrhoids reduced postoperative pain. Male and postoperative complications were identified as significant risk factors for incomplete resolution. Close follow-up monitoring of these high-risk patients is recommended to facilitate timely retreatment when necessary.

## Data Availability

The raw data supporting the conclusions of this article will be made available by the authors, without undue reservation.
